# Dietary Citrus Peel Supplementation Enhances Hepatic Energy Metabolism, Muscle 9-HODE Generation and Isoleucine Catabolism in Beef Cattle

**DOI:** 10.3390/metabo16030201

**Published:** 2026-03-18

**Authors:** Susumu Muroya, Koichi Ojima, Arata Banno, Hirotaka Nagai, Kazumasa Kakibuchi, Takuma Higuchi, Shuji Sakamoto, Kazutsugu Matsukawa

**Affiliations:** 1 Department of Animal Science and Welfare, Kagoshima University, Korimoto 890-0065, Kagoshima, Japan; 2 Division of Animal Products Research, NARO Institute of Livestock and Grassland Science (NILGS), Tsukuba 305-0901, Ibaraki, Japan; 3 Department of Agriculture and Marine Science, Kochi University, Nankoku 783-8502, Kochi, Japan; 4 Shikoku Research Institute Inc., Nishimachi, Takamatsu 761-0192, Kagawa, Japan; 5 Science Research Center, Kochi University, Nankoku 783-8505, Kochi, Japan

**Keywords:** bovine, citrus, fatty acid oxidation, metabolomics, postmortem aging, skeletal muscle

## Abstract

Background: Citrus components potentially suppress adipogenic differentiation and lipid accumulation, and exhibit anti-inflammatory and antioxidant effects. We hypothesized that the bioactive compounds in *Citrus junos* Sieb ex Tanaka (yuzu) fruit peel can alter the systemic metabolism and productivity of beef cattle. Methods: Japanese Brown (JBR) steers were fed with a diet supplemented with 2.5% yuzu peel during the last month of the finishing period. To investigate the effect of dietary yuzu supplementation (DYS) on beef and liver metabolism, we explored the metabolomic profiles of *longissimus thoracis* (LT, loin) muscle at 14 d postmortem using capillary electrophoresis (CE-TOF/MS) and high-performance liquid chromatography time-of-flight mass spectrometry (LC-TOF/MS). Results: The DYS treatment enhanced the beef fat score compared to that recorded in beef in the no-DYS (None) group (*p* = 0.050); however, the other carcass quality traits were not significantly different between the DYS and None groups. CE-TOF/MS and LC-TOF/MS revealed 242 and 107 annotated peaks, respectively, for the LT muscle. DYS significantly increased 9(*S*)-hydroxyoctadecadienoic acid (9-HODE, a beef flavor precursor), cyclo(-Leu-Pro), spermidine, asymmetric dimethylarginine, and 7α-hydroxycholesterol levels and reduced 2-ethylhydracrylic acid (2-EHAA), γ-tocopherol, coenzyme Q10 (CoQ10), sphingomyelin(d18:1/16:0), Cys-Gly, Tyr-Arg, and palmitoylcarnitine levels in postmortem LT muscle (*p* < 0.050). Concomitantly, in the fresh liver, DYS increased acetyl-CoA, 6-phosphogluconic acid, *S*-methylglutathione, ATP, ribulose 5-phosphate, and ADP levels and suppressed the content of thiamine, Ala-Ala, riboflavin, and ascorbate 2-sulfate (*p* < 0.050). Conclusion: Collectively, yuzu ingredients activated ATP production in the liver through the elevation of hepatic energy metabolism primarily in the citrate cycle and β-oxidation, and potentially altered muscle metabolism, including linoleic acid oxidation, FAD-mediated electron transport chain, and isoleucine catabolism, as demonstrated in the reduced accumulation of 2-EHAA and CoQ10 in DYS beef. Moreover, DYS likely affects the gut microbiome by enhancing the production of cyclo(-Leu-Pro), an antimicrobial dipeptide.

## 1. Introduction

Citrus fruits have multiple beneficial effects on human health when consumed as a plant-based food. Their dietary effects have been documented in studies investigating the health benefits of the Mediterranean diet [1,2,3], with several studies demonstrating reduced risks of cardiovascular disease (CVD) [4,5,6], diabetes [7,8], and cancers [9]. Various types of citrus fruits are enriched in bioactive compounds [5,10,11], such as carotenoids, terpenes, limonoids, and flavonoids. Accumulating evidence suggests that citrus flavonoids, including flavones, flavanones, flavonols, isoflavones, anthocyanidins, and flavanols [12,13], exert antioxidant [13], hypolipidemic [14], hypoglycemic [15,16], and anticarcinogenic bioactivities [17,18]. Dietary citrus flavonoids evoke their functions to modulate systemic glucose and lipid metabolism, exerting hyperlipidemic and hyperglycemic effects in humans and animals [19,20].

The positive effects on human health are not limited to the edible citrus fruits but have also been detected in citrus processing waste [21,22]. Citrus peels contain major beneficial flavonoid compounds, including naringin, hesperidin, and nobiletin, which exhibit bioactivities comparable to those of fruits [23,24]. Animal model experiments have demonstrated the hypoglycemic, hypocholesterolemic, and antioxidant effects of naringin [25,26,27,28,29], thereby reducing obesity risks [30,31]. Hesperidin also exhibits hyperlipidemic, hyperglycemic, and antioxidant activities [32,33,34,35]. Most of these activities, as well as the stimulating effect of adipose thermogenesis, are shared by naringin and hesperidin [31,36]. Furthermore, nobiletin has been reported to alter liver metabolism [37], leading to the amelioration of hepatic steatosis [38] and enhancement of skeletal muscle mass [39]. These studies further suggest a broad-range impact of nobiletin on the modulation of gut microbiota and homeostatic metabolism [37,40].

In animal science and industries, citrus waste has been highlighted as a dietary supplement owing to its potential benefits in improving animal growth performance and meat quality. The beneficial effects of citrus pulp are likely associated with various ingredients, such as naringin and hesperidin, which alter the expression of genes involved in antioxidant activity [41] and lipid metabolism [42,43]. High proportions (24–35%) of dried citrus pulp in a concentrate-based diet altered the fatty acid composition in lamb [44] and ostrich muscles [45] and improved meat oxidative stability in lambs [46]. In addition, citrus pulp-supplemented diets improve beef quality, sensory attributes [47,48] and oxidative stability [49,50,51]. However, the effect of citrus waste administration on metabolic alteration in beef cattle remains inadequately characterized. Previous reports inspired us to hypothesize that dietary citrus peel supplementation alters hepatic lipid and skeletal muscle metabolism in beef cattle.

Therefore, we investigated citrus peel-supplemented diet-induced variation in hepatic and skeletal muscle metabolism in Japanese Brown (JBR) cattle, a minor population breed with moderately marbled beef and high growth performance [52,53,54]. In this study, we used the peel of *Citrus junos* Sieb ex Tanaka (yuzu) as a citrus-based supplement with known antioxidant activity [55,56]. The postmortem aged beef and fresh livers of cattle fed the yuzu peel-supplemented diet were analyzed using capillary electrophoresis (CE-TOF/MS) and/or high-performance liquid chromatography time-of-flight mass spectrometry (LC-TOF/MS) to obtain comprehensive metabolite profiles.

## 2. Materials and Methods

### 2.1. Animals

Six JBR steers (Kochi [Tosa] pedigree [JBRT]) were fed and reared at Kochi University farm until 28 months of age. The basic individual diets were designed based on energy and nutrient requirements according to the standard diet model of the Japanese Feeding Standard for Beef Cattle (JFSBC, 2008 ed.) [57] following a previously described method [52]. The cattle were assigned to two dietary treatment groups (*n* = 3 for each): treated with (DYS) or without (None) yuzu supplementation. Supplementation was provided as 2.5% (*w*/*w*) of yuzu peel during the last month of the finishing period. Beef quality was evaluated using dressed carcasses at a commercial slaughterhouse (Meat Pro Kochi, Kochi, Japan), according to a standard determined by the Japan Meat Grading Association (https://www.jmga.or.jp/; 9 September 2023).

### 2.2. Muscle and Liver Samples

Fresh liver samples were collected immediately after the slaughter. The *longissimus* muscle (LT) was stored for 14 days at 2 °C after slaughter. From the postmortem-aged muscle, lean portions of small LT muscle pieces were carefully collected from multiple locations in the muscle. Liver and muscle samples were stored at −80 °C until use.

### 2.3. CE-TOF/MS Measurement

Samples for CE-TOF/MS measurements were prepared as previously described [52,58]. For this analysis, frozen muscle pieces (approximately 27.7–38.5 mg) were rapidly immersed in 50% acetonitrile containing 10 μM Internal Standard Solution 1 (Human Metabolome Technologies (HMT), Tsuruoka, Japan) and homogenized at 0 °C, followed by centrifugation at 2300× *g* for 5 min at 4 °C. Subsequently, the aqueous phase was filtered using a 5 kDa cutoff membrane. The lyophilized filtrate was suspended in Milli-Q water and analyzed using an Agilent CE capillary electrophoresis system (Agilent Technologies, Waldbronn, Germany), while maintaining the analytical conditions consistent with those used in a previous study [58].

### 2.4. LC-TOF/MS Measurement

For LC-TOF/MS analysis, the frozen muscle pieces (25.2–45.4 mg) were immediately soaked in 1% formic acid/acetonitrile solution containing internal standards (H3304-1002, HMT). Subsequently, the samples were crushed three times under frozen conditions for 120 sec at 1500 rpm, followed by one crushing cycle after the addition of 1% formic acid solution and a final cycle after the addition of Milli-Q water. Following a centrifugation step (2300× *g*, 5 min, 4 °C), the upper layer solution was filtered using a 3 kDa cutoff filter (NANOCEP 3k OMEGA, PALL Corporation, Ann Arbor, MI, USA) to remove macromolecules and subsequently filtered through a Hybrid SPE-Phospholipid cartridge (Hybrid SPE-Phospholipid 30 mg/mL, SUPELCO, Bellefonte, PA, USA). The lyophilized filtrate was suspended in 50% isopropanol aqueous solution and analyzed by LC-FT/MS. Metabolomic analyses were performed using an Agilent 1260 series RRLC system SL (Agilent Technologies, Waldbronn, Germany) combined with an Agilent LC/MSD TOF MS system (Agilent Technologies, Waldbronn, Germany). The conditions (e.g., flow rate and gradient conditions) for metabolite separation using an octadecylsilyl (ODS) column were set following a previous report for cation and anion modes [53]. ESI-MS was conducted in the positive and negative ion modes, with capillary voltages of 4000 and 3500 V for cationic and anionic mode analyses, respectively. The spectrometer scanned from 100 to 1700 *m*/*z*.

### 2.5. Data Analysis of MS Measurement Results

Raw CE-TOF/MS and LC-TOF/MS data were analyzed using MasterHands software (ver. 2.19.0.2) using a previously described method [53]. For this analysis, the signal peaks of isotopomers, adduct ions, and other product ions of known metabolites were removed, all signal peaks of interest were extracted, and their migration times (MTs) or retention times (RTs) were normalized to those of the internal standards. Alignment of the peaks was performed, and the detected peaks were annotated using the HMT metabolite database. To analyze the relative compound levels between the feeding treatments, the peak areas were further normalized using the sample weight. In the comparative analysis, the abundance of undetected compounds was considered zero.

### 2.6. Statistical Analyses

The differences in daily gain (DG), final body weight (BW), carcass weight, rib eye area (REA), and subcutaneous fat thickness (SFT) between the dietary treatments were tested using the Welch’s *t*-test, whereas variations in beef marbling, color, and fat scores were assessed using the Mann–Whitney U-test. Relative content values were analyzed using MetaboAnalyst (https://www.metaboanalyst.ca/; 26 February 2024), based on principal component analysis (PCA), hierarchical cluster analysis (HCA), Student’s *t*-test, and metabolite set enrichment analysis (MSEA). The differences between treatments with and without DYS were considered statistically significant at *p* < 0.050, with a difference trend at 0.050 ≤ *p* < 0.100. In the OPLS-DA score plot, the confidence level of the ellipse was 95%.

## 3. Results

### 3.1. Result of Carcass Evaluation

The effects of the DYS treatment on beef cattle carcasses are presented in Table 1. Most of the quality traits of carcasses were not affected by DYS; however, the beef fat score, primarily determined by fat color and gloss, was higher in beef supplemented with yuzu than in the None group (*p* = 0.050).

### 3.2. Effect of DYS on the JBR Beef Metabolome

To understand the effects of DYS on the JBR beef quality and postmortem metabolism, we profiled beef metabolites. In total, 242 and 107 annotated peaks were detected using CE-TOF-MS and LC-TOF-MS, respectively, across all beef samples examined (Supplementary Tables S1 and S2).

**Figure 1:**
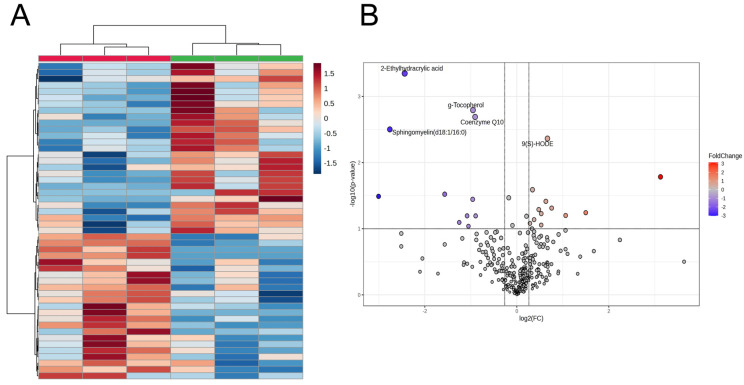
Heatmap result of HCA (**A**) and volcano plot (**B**) of metabolite profile data from CE-TOF/MS and LC-TOF/MS metabolomics profiles of JBR beef with and without DYS. In the HCA, the results of statistically representative top 50 metabolites were used. Subject means individual animal. The beef samples with and without DYS are grouped with green and red bars, respectively.

The CE-TOF/MS and LC-TOF/MS beef metabolome profiles were then integrated, and the data were analyzed using multivariate methods with HCA and PCA. The HCA of aged LT muscle samples revealed variations in highly different 50 metabolites between the DYS and None groups (Figure 1), which were attributed to the characteristic distribution of representative beef metabolites and their abundance in beef samples. In both the HCA heatmap and volcano plot, suppression of 2-ethylhydracrylic acid (2-EHAA), γ-tocopherol, Coenzyme Q10 (CoQ10), and sphingomyelin (d18:1/16:0) and an increase in 9(*S*)-hydroxyoctadecadienoic acid (9(*S*)-HODE) in DYS beef highly contributed to the beef sample separation between DYS treatments (Table 2). On the other hand, the beef samples were not distinguished between the two groups based on the metabolomics profile in PCA (Supplementary Figure S1). In addition, the levels of dipeptide cyclo(-Leu-Pro) (cLP), spermidine, asymmetric dimethylarginine (ADMA), and 7α-hydroxycholesterol (7α-OHCh) were higher in the DYS group than in the control group, whereas Cys-Gly, Tyr-Arg, and palmitoylcarnitine (PALCAR) were reduced (*p* < 0.050, Table 2). Acylcarnitine(14:0) (*p* = 0.080) and acylcarnitine(18:1) (*p* = 0.092) were less abundant in the DYS group than in the control group.

**Figure 2:**
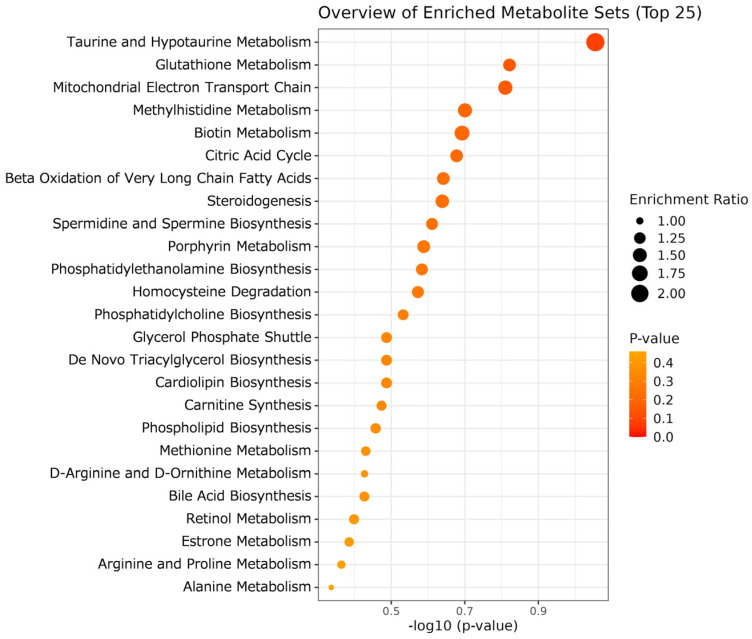
MSEA results for metabolomic difference in aged beef with and without DYS. Enrichment ratio is computed by (observed hits)/(expected hits).

MSEA was performed to understand the metabolic mechanisms underlying the effects of DYS on beef. For beef analysis, we used data from a set of 310 metabolites, which included the most abundant single isomer of each lipid metabolite, with unidentified steric isomers. As shown in Figure 2, among the top 25 differentially abundant metabolites between the two dietary treatments, only Taurine and Hypotaurine Metabolism exhibited a trend of difference in the DYS group (*p* = 0.088). Therefore, although aged LT muscle metabolism was not likely to be modulated, several lipids and metabolites linked to oxidative phosphorylation (OXPHOS), such as CoQ10 and 9(*S*)-HODE, were affected by DYS (Figure 1).

### 3.3. Effect of DYS on the JBR Liver Metabolome

Since DYS affects specific lipids and the oxidation-associated metabolite CoQ10, we hypothesized that DYS affects energy metabolism in the liver, the major tissue involved in CoQ10 biosynthesis [59], which potentially modulates skeletal muscle metabolism. Accordingly, we analyzed the metabolomic profiles of fresh livers collected from steers in the DYS and None groups using CE-TOF/MS to focus on energy metabolism-associated metabolites. In this analysis, 308 annotated peaks were detected across all tested liver samples (Supplementary Table S3).

**Figure 3:**
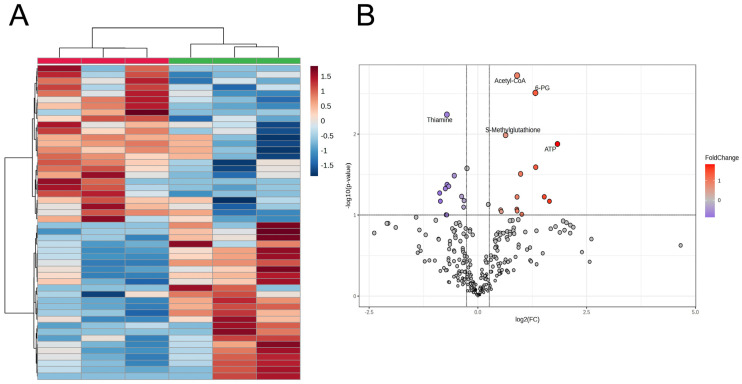
Heatmap result of HCA (**A**) and volcano plot (**B**) of metabolite profile data from CE-TOF/MS metabolomics profiles of JBR fresh liver with and without DYS. In the HCA, the results of statistically representative top 50 metabolites were used. Subject means individual animal. The beef samples with and without DYS are grouped with green and red bars, respectively.

The HCA results for highly different 50 metabolites revealed clear discrimination of the liver samples based on dietary treatments (DYS and None) (Figure 3). Acetyl-CoA, 6-phosphogluconate (6-PG), *S*-methylglutathione (MGSH), and ATP levels were higher, and thiamine levels were reduced in the DYS liver more than in its None counterpart (Figure 3, Table 3). Although the liver samples were not distinguished between the DYS and None groups by the metabolomics profile in PCA (Supplementary Figure S2), higher levels of ribulose 5-phosphate (Ru5P) and Ala-Ala and lower levels of ADP, riboflavin, and ascorbate 2-sulfate (ascorbate 2-S) in the DYS group compared with those in the control group (*p* < 0.050) were also observed (Table 3) in addition to the five representative metabolites. Furthermore, glutamic acid (Glu, *p* = 0.059), glucose 1/6-phosphate (G1P/G6P, *p* < 0.100), and citric acid (*p* = 0.091) showed a trend of higher levels in the DYS group than in the None group, while succinic acid was at a lower level (*p* = 0.047) (Table 3).

**Figure 4:**
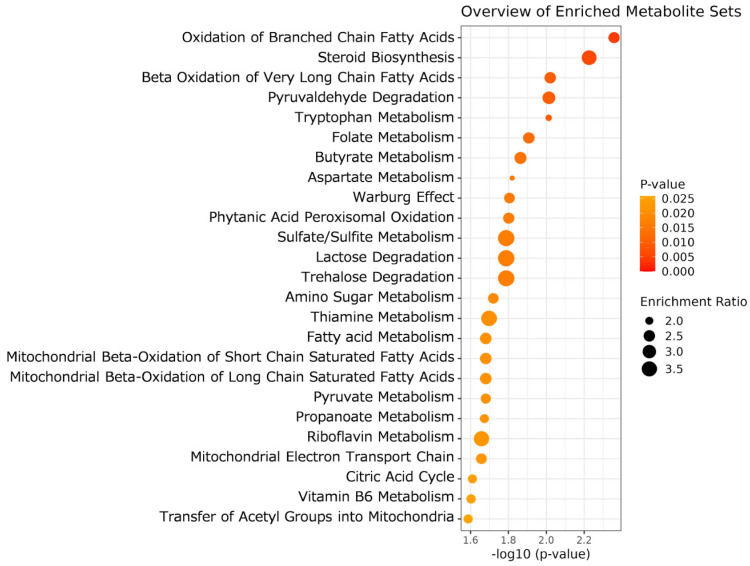
MSEA results for metabolomic difference in fresh liver with and without DYS. Enrichment ratio is computed by (observed hits)/(expected hits).

Furthermore, we performed MSEA focusing on the liver metabolome profile, comprising 287 metabolites. The top 25 liver metabolic processes depicted in Figure 4 differed significantly between the two dietary treatments (*p* < 0.050). These representative metabolisms were mainly associated with fatty acid oxidation in mitochondria (e.g., β-oxidation, acetyl group transfer, electron transport chain) and energy substrate metabolisms (e.g., metabolisms of propanoate, pyruvate, citrate). The DYS steer liver samples also exhibited alterations in the steroid synthesis and metabolism of tryptophan, folate, butyrate, aspartate, sulfur compounds, thiamine, and riboflavin. These results indicate that DYS significantly affected fatty acid oxidation, the tricarboxylic acid (TCA) cycle, and mitochondrial OXPHOS in the liver of JBR steers.

## 4. Discussion

The present study revealed the DYS-induced alteration in the metabolome profile in aged beef, which was particularly associated with lipid metabolism and OXPHOS, such as γ-tocopherol, CoQ10, and 9-HODE. The results suggest that DYS led to lipolysis but suppressed lipogenesis, as demonstrated by previous *in vivo* and *in vitro* studies on citrus fruits and peels [60,61,62]. The anti-lipogenic effects are potentially driven by the major functional flavonoids, such as naringin and hesperidin, derived from citrus tissues via oxidative [29,63], thermogenic metabolic pathways [64], or β-adrenergic receptor signaling [65,66,67].

### 4.1. Lipid Oxidation

As shown in Figure 1B, 9-HODE levels in JBR beef were significantly higher in the DYS group than in the None group. Intriguingly, 9-HODE was recently reported to be the primary precursor of γ-hexalactone [68]. Lactones, including γ-hexalactone, are generated as key volatile compounds associated with the “sweet beef aroma” from linoleic acid in thermal-cooked beef from Japanese Black (Wagyu) cattle [69,70], a world-renowned breed characterized by high intramuscular fat (marbling) content and sweety aroma. Although JBR beef, including that examined in this study, exhibits a lower level of marbling than that observed in the Wagyu beef, the JBR beef fed DYS might have a stronger sweet aroma than beef fed without DYS supplementation.

The original 9-HODE content in citrus and its generation from other lipids after dietary intake in beef remain inadequately understood. The synthesis of 9-HODE is enhanced by the oxidation of fatty acids, primarily linoleic acid [71,72], in oxidized low-density lipoproteins (LDL) [73]. Moreover, triglyceride-rich lipoprotein lipolysis increases the 9-HODE level [74]. In this study, the decline in Cys-Gly, the degradation product of glutathione, and a trend of elevated oxidized glutathione (GSSG) content (*p* < 0.100; Table 2) potentially reflect that DYS modulates the oxidative microenvironment in aged beef tissues. Accordingly, linoleic acid in the muscle tissue is potentially more prone to oxidation, leading to 9-HODE formation, in DYS steers than in non-DYS steers. Therefore, yuzu peel ingredients likely contributed to the enhancement of linoleic acid oxidation; however, further investigation can clarify the mechanism underlying this oxidation.

In terms of lipid oxidation, an increase in 7α-OHCh was in line with that in 9-HODE (Table 2). 7α-OHCh, a primary oxidation product in the 7α-hydroxylation of cholesterol, is both enzymatically and non-enzymatically (by free radical oxidation) formed. The enzymatic reaction is catalyzed by cholesterol 7α-hydroxylase (CYP7A1) [75], which is a rate-limiting step of bile acid synthesis under most conditions [76]. Since both linoleic acid and cholesterol are loaded onto LDL and transported from the liver to recipient tissues through circulation, we consider that both compounds might be derived from the liver and transported by LDL; the abundance of linoleic acid in human LDL corroborated this fact [77]. Collectively, the increase in linoleic acid and cholesterol oxidation products supports the hypothesis that DYS upregulated lipid oxidation in the DYS group. Furthermore, these changes in muscle fatty acid metabolism potentially contributed to the improvement in beef fat score, especially promoting the color and gloss, by altering lipid composition.

The increase in ADMA levels in aged beef also suggests that DYS induces lipid oxidation in the LT muscle (Table 2). ADMA, an endogenous inhibitor of nitric oxide (NO) synthase, is generated by arginine *N*-methyltransferase (PRMT). Oxidized LDL, enriched in oxidized lipids, induces PRMT expression in endothelial cells [78,79]. Overall, the increase in ADMA in DYS beef was likely triggered by lipid oxidation in the muscle.

Macrophages in muscle or intramuscular fat tissue may also be considerably involved in 9-HODE generation because 9-HODE is generated from linoleic acid by arachidonic acid 15-lipoxygenase (ALOX15) in macrophages at the onset of the proinflammatory process [80]. The increasing trend in histamine levels observed in this study (Table 2; *p* = 0.051) was in line with the context of pro-inflammation. 9-HODE is a ligand of endogenous peroxisome-proliferator-activated receptor γ (PPARγ) and regulates macrophage gene expression through PPARγ [73]. Moreover, 9-HODE generated in the tissue macrophages induced the differentiation of preadipocytes into brown/beige adipocytes [80], indicating its role in the non-inflammatory removal of apoptotic white adipocytes during adipose remodeling. Interestingly, orange juice [81], which contains hesperidin [82], potentially exhibits anti-inflammatory effects. These results suggest that yuzu ingredients elicit lipid oxidation, leading to 9-HODE generation in tissue macrophages in bovine muscle. This might accompany feedback activation of macrophages through PPARγ, which might result in alteration of adipose deposition in skeletal muscle tissue of DYS steers.

Furthermore, the potential yuzu ingredients nobiletin, naringin, and hesperidin might directly regulate expression of *PPARG*, peroxisome-proliferator-activated receptor α (*PPARA*), and PPARG coactivator 1 α (*PGC1A*) in the marbling fat depot in DYS beef tissue. Particularly, naringin has a crucial impact on adipogenesis and fatty acid oxidation through downregulation of *PPARG* and upregulation of *PPARA* and *PGC1A* [64]. Since expression or activity of these genes remain unknown due to a lack of fresh muscle samples in this study, further studies are required to elucidate mechanisms on how dietary yuzu ingredients alter metabolism through transcriptional regulation.

### 4.2. FAD-Mediated Muscle Redox Metabolism and Isoleucine Catabolism

Intriguingly, the levels of CoQ10, PALCAR, and 2-EHAA decreased in DYS-aged beef (Table 2). 2-EHAA is normally present in urea at low levels as a catabolic end-product; however, it accumulates when isoleucine catabolism is impaired [83]. Given the reduced 2-EHAA levels (Figure 1B, Table 2), it is likely that isoleucine catabolism was enhanced in DYS beef through modulated activity of short/branched-chain-specific acyl-CoA dehydrogenase (SBCAD).

**Figure 5:**
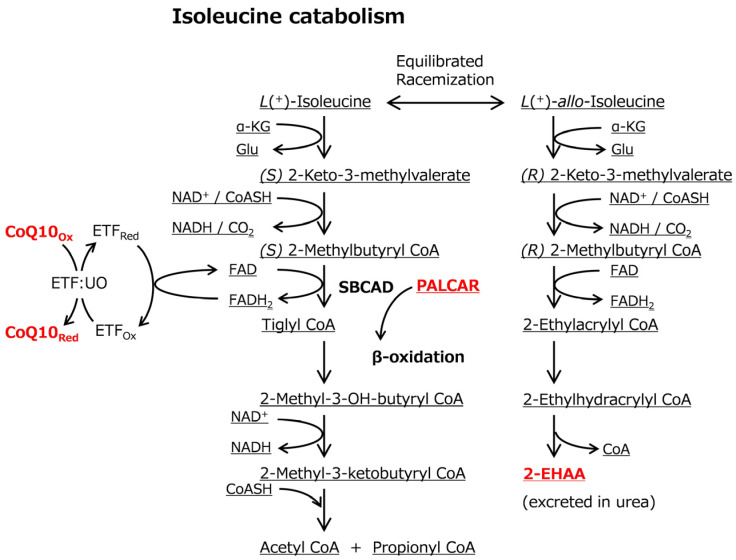
Hypothetic scheme that shows representative muscle metabolites (red) and microenvironment metabolisms affected by DYS in this study. Isoleucine catabolism is tightly linked not only with β-oxidation and FAD redox reaction in ETC but also with 2-EHAA generation. α-KG: α-ketoglutarate; FTF:UO: electron transfer flavoprotein-ubiquinone oxidoreductase.

Deficiency of SBCAD activity is a well-known cause of the accumulation of 2-EHAA in urine and plasma [83]. Isoleucine is normally catabolized to (*S*) 2-methylbutyril CoA (2-MB CoA), generating acetyl-CoA and propionyl-CoA (Figure 5). During isoleucine catabolism, SBCAD dehydrogenates (*S*) 2-MB CoA to tiglyl-CoA, reducing flavin adenine dinucleotide (FAD) via a conjugation reaction [84]; thereby, it is involved in β-oxidation and amino acid catabolism. However, the inhibition of SBCAD activity, as illustrated in individuals with mutations in *ACADSB*, is associated with the accumulation of (*S*) 2-MB CoA and other intermediates, which affects racemic equilibration between isoleucine isomers and consequently leads to accumulation of 2-EHAA (Figure 5). In this study, as 2-EHAA levels decreased (Figure 1B, Table 2), DYS likely enhanced isoleucine catabolism rather than the *allo*-isoleucine pathway, which could be associated with upregulated SBCAD activity.

The modulated isoleucine catabolism was likely connected with alteration of CoQ10 and PALCAR by SBCAD, the key conjugating enzyme; however, it remains unclear how these β-oxidation-related metabolites were associated with the enhanced isoleucine catabolism. CoQ10, acting as an electron pool in electron transport chain (ETC), promotes phosphorylation of AMP-activated protein kinase (AMPK) and fatty acid oxidation (β-oxidation) [83,85,86]. In cases of aberrant SBCAD activity, FAD re-oxidation in the electron transfer flavoprotein (ETF)-catalyzed reaction can be modulated, potentially affecting the biosynthesis of CoQ10, because it is tightly connected to the redox reaction of FAD (Figure 5). Accordingly, CoQ10 and PALCAR levels might be affected by low availability of SBCAD activity due to the enhanced isoleucine catabolism, altered redox metabolisms, or modulated CoQ10 supply from liver. The redox reactions linked to the ETC may be associated with altered levels of GSSG and the degradation product Cys-Gly (*p* < 0.100; Table 2). Taken together, yuzu ingredients might have a different impact on isoleucine catabolism and β-oxidation in postmortem bovine muscle.

Given that β-oxidation and isoleucine catabolism were primarily confined to muscle cells, the phenotypic metabolite changes in DYS beef could be a result of altered gene expression of *ACADSB* in addition to AMPK genes. Based on the reduced muscle acylcarnitines and increased hepatic acetyl-CoA in DYS steers, the systemic energy was at a high level, suggesting that expression of *ACADSB* and/or the upstream genes might be upregulated.

cLP is generated by various bacteria and fungi, including gut microorganisms, but not by mammalian cells. Hence, it is occasionally found in food materials [87] and plays a role in the defense against other microorganisms and in growth suppression. Recently, cLP has been recognized as an anti-cariogenic and antifungal agent. Therefore, an increased level of cLP in DYS beef (Table 2) may improve the shelf life of beef; however, the mechanism underlying the DYS-induced increase in cLP content remains unclear. Microbiome analysis is necessary to investigate the potential contribution of changes in gut microbiota.

### 4.3. Liver Metaboilsm

DYS also altered hepatic metabolism, including the oxidation of branched-chain and very long-chain fatty acids, as demonstrated through MSEA (Figure 4). Particularly, in the liver, riboflavin levels decreased following DYS treatment. This might affect FAD metabolism and further modulate FAD redox-linked β-oxidation catalyzed by SBCAD and other Acyl-CoA dehydrogenases (ACDHs), consistent with our observation of significant metabolic alterations in mitochondrial β-oxidation, ETC, and acetyl group transfer (Figure 4). Cumulatively, β-oxidation in the liver was likely modulated by DYS. In contrast to β-oxidation, enhanced use of alternative energy substrates is suggested by the upregulated ATP and 6-PG generation (*p* < 0.050). Nevertheless, further investigation is required to elucidate the mechanisms underlying the alteration of energy metabolism in DYS steer livers. In the livers of DYS steers, acetyl-CoA (*p* = 0.002), G1P, G6P, and citric acid (*p* < 0.100) exhibited increasing trends, whereas succinic acid showed a reduced level (*p* = 0.047; Table 3), potentially indicating the downregulation of glycolysis and the TCA cycle, followed by accumulation of the glycolytic substrate G1P, which is possibly attributable to the accumulation of acetyl-CoA and citric acid. Alterations in energy metabolism are potentially linked to altered β-oxidation. Previous animal model-based research demonstrated that citrus ingredients, such as naringin, hesperidin, and nobiletin, exhibit multiple effects, particularly on energy metabolism, which include hypoglycemic and hypocholesterolemic improvement [25,26,27,28,29,37]. Accordingly, yuzu peel ingredients might modulate glycolysis and the TCA cycle in addition to β-oxidation in DYS steer liver. Further studies can clarify the underlying modulation of liver metabolisms. Naringin suppresses adipogenesis by downregulation of *PPARG* and enhances fatty acid oxidation by upregulation of *PPARA*, leading to the improvement of impaired lipid metabolism in a model of nonalcoholic fatty liver disease (NAFLD) [88]. Accordingly, yuzu ingredients including naringin might modulate the expression of adipogenesis regulatory genes in DYS liver.

Amino acid metabolites, such as tryptophan and aspartate, were also extracted in addition to valine, leucine, and isoleucine degradation (Figure 4). The analysis of aged DYS-treated beef samples reflected that isoleucine catabolism in the liver was affected by DYS in an FAD-associated manner. MGSH, a metabolite upregulated in the DYS-treated liver (*p* = 0.010), is generated by methylation of the SH group of glutathione, a major endogenous antioxidant [89]. Analysis using diabetic rats chronically administered *C. unshiu* extracts demonstrated that MGSH increased in the livers of mice fed dietary supplementation with *Citrus tumida hort. ex Tanaka*, suggesting that *C. tumida* reduces oxidative stress in hepatic tissues [90].

### 4.4. Limitations

In this study, three steers were assigned to each treatment, which constitutes a minimal number for the experimental design. Nonetheless, our findings reflecting the overall differences in metabolite profiles and metabolisms were in line with our previous reports on post-mortem aged beef. Hence, we conclude that the essential trend of metabolite distribution and differences observed in the current results appear consistent with the outcomes of the studies using larger numbers of animals; however, the results need to be confirmed through large-scale analyses in the future.

## 5. Conclusions

The present study demonstrated that DYS altered the metabolism of post-mortem aged beef and fresh livers. In beef, we detected alterations in 2-EHAA, PALCAR, and CoQ10 levels. Based on the metabolite network connecting these metabolites, the results suggest that DYS substantially modulated isoleucine catabolism and β-oxidation, which are associated with FAD redox and SBCAD activity. DYS also enhanced 9-HODE, a favorable flavor precursor in beef, indicating yuzu ingredient-mediated enhancement of linoleic acid oxidation. Moreover, DYS modulated hepatic metabolites in steers, particularly ATP and energy metabolism intermediates, which might be associated with changes in beef metabolites through circulation.

## Figures and Tables

**Table 1:** Beef quality traits of JBR steers finished with/without dietary yuzu supplementation (DYS).

		None		DYS		
Quality Traits		Mean/Score	S.D.	Mean/Score	S.D.	*p*-Value
Finishing and carcass results		Mean		Mean		
	Daily gain (kg/d)	0.57	0.13	0.58	0.23	0.947
	Final body weight (kg)	699.33	18.55	724.33	40.94	0.476
	Carcass weight (kg)	433.33	7.39	469.27	13.40	0.191
	Rib eye area (cm^2^)	46.67	5.79	50.33	5.25	0.543
	Subcutaneous fat thickness (cm)	2.93	1.28	2.07	0.33	0.407
Grading score		Score		Score		
	Beef marbling (1–12)	3, 4, 3	-	2, 3, 3	-	0.302
	Beef color (1–5)	5, 4, 5	-	5, 5, 5	-	0.700
	Beef fat (1–5)	3, 2, 2	-	4, 4, 4	-	0.050

S.D.: standard deviation.

**Table 2:** Differently altered metabolites by dietary yuzu supplementation (DYS) in beef (*p* < 0.100).

Metabolite	Fold Change (DYS/None)	*p*-Value
2-Ethylhydracrylic acid	0.18	<0.001
γ-Tocopherol	0.52	0.002
Coenzyme Q10	0.53	0.002
Sphingomyelin(d18:1/16:0)	0.15	0.003
9(*S*)-hydroxyoctadecadienoic acid	1.59	0.004
Cyclo(-Leu-Pro)	8.72	0.016
Spermidine	1.27	0.026
Cys-Gly	0.34	0.030
Tyr-Arg	0.12	0.032
Palmitoylcarnitine	0.51	0.036
Asymmetric dimethylarginine	1.55	0.039
7α-Hydroxycholesterol	1.69	0.049
Histamine	1.39	0.051
Pyrraline	2.83	0.057
Oxidized glutatihone (GSSG)	1.45	0.059
*N*ω-Methylarginine	2.09	0.063
Isoglutamic acid	0.47	0.064
Cortisol	0.54	0.064
Ethanolamine phosphate	1.30	0.073
Acylcarnitine(14:0)	0.42	0.080
Glycine	1.22	0.083
Linoleyl ethanolamide	1.44	0.088
Acylcarnitine(18:1)	0.48	0.092

**Table 3:** Differently altered metabolites by dietary yuzu supplementation (DYS) in liver (*p* < 0.100).

Metabolite	Fold Change (DYS/None)	*p*-Value
Acetyl-CoA	1.87	0.002
6-Phosphogluconic acid	2.50	0.003
Thiamine	0.61	0.006
*S*-Methylglutathione	1.55	0.010
ATP	3.55	0.013
Ribulose 5-phosphate	2.51	0.026
ADP	1.98	0.031
Ala-Ala	0.69	0.033
Riboflavin	0.61	0.042
Ascorbate 2-sulfate	0.63	0.044
Succinic acid	0.60	0.047
Pantothenic acid	0.54	0.054
Glutamic acid	0.77	0.059
GDP	2.88	0.060
ADP-ribose	1.87	0.060
4-Oxopyrrolidine-2-carboxylic acid	0.80	0.066
Putrescine	0.55	0.067
CMP-*N*-acetylneuraminate	3.13	0.067
5-Hydroxylysine	0.80	0.080
Glucose 1-phosphate	1.86	0.085
Sarcosine	1.44	0.086
Glucose 6-phosphate	1.87	0.089
Citric acid	1.45	0.091

## Data Availability

Data are contained within the article or Supplementary Materials (Tables S1–S3). The data presented in this study are available at doi.org/10.5281/zenodo.18592160.

## Supplementary Materials

The following supporting information can be downloaded at: https://www.mdpi.com/article/10.3390/metabo16030201/s1, Figure S1: PCA Score plot of beef samples, Figure S2: PCA Score plot of liver samples. Table S1: Detected features and information on the identification of the annotated peaks in CE-TOF/MS analysis of beef samples, Table S2: Detected features and information on the identification of the annotated peaks in LC-TOF/MS analysis of beef samples, Table S3: Detected features and information on the identification of the annotated peaks in LC-TOF/MS analysis of liver samples.
