# Supplementary material for: Dietary Citrus Peel Supplementation Enhances Hepatic Energy Metabolism, Muscle 9-HODE Generation and Isoleucine Catabolism in Beef Cattle
Source: Metabolites. 2026 Mar 18;16(3):201. doi: 10.3390/metabo16030201 (PMC13028232; doi:10.3390/metabo16030201)

Supplementary figure S1. PCA Score plot of beef samples.

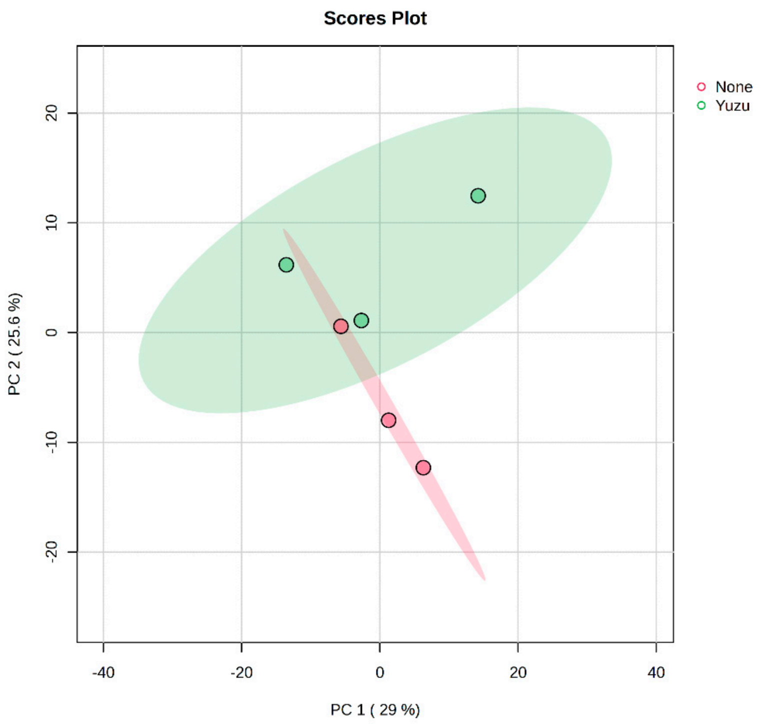

Supplementary figure S2. PCA Score plot of liver samples.

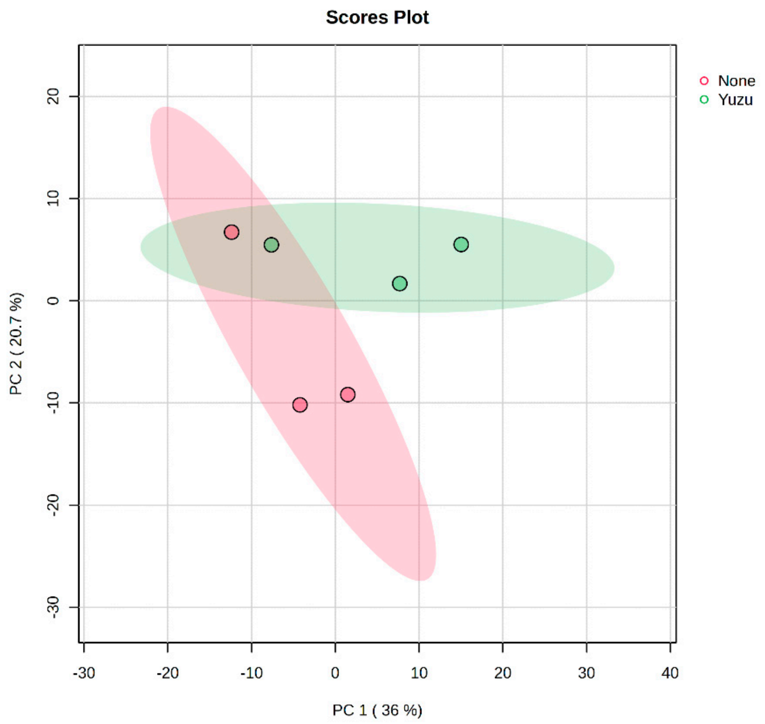

Supplement: Supplementary file 1 [file metabolites-16-00201-s001.zip › Supplementary figures.pdf]
